# Long-term nephrostomy in an adult male spinal cord injury patient who had normal upper urinary tracts but developed bilateral hydronephrosis following penile sheath drainage: pyeloplasty and balloon dilatation of ureteropelvic junction proved futile: a case report

**DOI:** 10.1186/1757-1626-2-9335

**Published:** 2009-12-16

**Authors:** Subramanian Vaidyanathan, Bakul M Soni, Peter L Hughes, Gurpreet Singh, Paul Mansour, Tun Oo

**Affiliations:** 1 Spinal Injuries Unit, District General Hospital, Town lane, Southport PR8 6PN, UK; 2 Department of Radiology, District General Hospital, Southport PR8 6PN, UK; 3 Department of Urology, District General Hospital, Southport PR8 6PN, UK; 4 Department of Cellular Pathology, District General Hospital, Southport PR8 6PN, UK

## Abstract

**Introduction:**

The consequences of spinal cord injury upon urinary bladder are readily recognised by patients and health care professionals, since neuropathic bladder manifests itself as urinary incontinence, or retention of urine. But health care professionals and persons with spinal cord injury may not be conversant with neuropathic dysmotility affecting the ureter and renal pelvis. We report an adult male patient with spinal cord injury, who developed bilateral hydronephrosis after he started managing neuropathic bladder by penile sheath drainage.

**Case presentation:**

A male patient, born in 1971, sustained spinal cord injury following a motorbike accident in September 1988. In November 1988, intravenous urography showed normal upper tracts. He was advised spontaneous voiding with 2-3 catheterisations a day. In February 1995, this patient developed fever, chills and vomiting. Blood urea: 23.7 mmol/L; creatinine: 334 umol/L. Ultrasound revealed marked hydronephrosis of right kidney and mild hydronephrosis of left kidney. Bilateral nephrostomy was performed in March 1995. Right pyeloplasty was performed in May 1998. In July 2005, this patient developed urine infection and was admitted to a local hospital with fever and rigors. He developed septicaemia and required ventilation. Ultrasound examination of abdomen revealed bilateral hydronephrosis and multiple stones in left kidney. Percutaneous nephrostomy was performed on both sides. Subsequently, extracorporeal shock wave lithotripsy of left renal calculi was carried out. Right nephrostomy tube slipped out in January 2006; percutaneous nephrostomy was performed again. In June 2006, left ureteric antegrade stenting was performed and nephrostomy tube was removed. Currently, right kidney is drained by percutaneous nephrostomy and left kidney is drained by ureteric stent. This patient has indwelling urethral catheter.

**Conclusion:**

It is possible that regular intermittent catheterisations along with anticholinergic medication right from the time of rehabilitation after this patient sustained paraplegia might have prevented the series of urological complications. Key components to successful management of external drainage of kidney in this patient are: [1] use of size 14 French pigtail catheter for long-term nephrostomy, [2] anchoring the catheter to skin to with Percufix catheter cuff to prevent accidental tug [3], replacing the nephrostomy dressing once a week by the same team in order to provide continuity of care, and [4] changing nephrostomy catheter every six months by a senior radiologist.

## Introduction

Pyeloureteral tract receives its innervation mainly by unmyelinated fibres, which originate from the renal, ovarian/spermatic, and sympathetic plexuses. The lower part of the ureter may receive additional pelvic innervation. The sympathetic supply to the ureter arises from T11-L1 spinal segments. At least part of these fibres synapses in the distal pole of the inferior mesenteric ganglion [1]. The consequences of spinal cord injury upon urinary bladder are readily recognised by patients and health care professionals, as clinical presentation of neuropathic bladder is very obvious in terms of urinary incontinence, or retention of urine. But health care professionals and spinal cord injury patients may not be conversant with neuropathic dysmotility affecting the ureter and renal pelvis. We report an adult male patient, who developed bilateral hydronephrosis after he started managing neuropathic bladder by penile sheath drainage. Impaired drainage of urine from renal pelvis due to neuropathic dysmotility contributed to development of bilateral hydronephrosis, which manifested clinically as severe urinary sepsis. Initially, percutaneous nephrostomy was performed as an emergency procedure. Later we attempted to improve drainage from renal pelvis by performing pyeloplasty. As pyeloplasty was unsuccessful, balloon dilatation of pelvi-ureteric junction was performed, which was also futile. In hindsight, we recognised the futility of carrying out these procedures, as pyeloplasty and balloon dilatation of pelviureteric junction were aimed solely to improve mechanical aspects of urinary drainage. These procedures did not correct the underlying pathology, which was neuropathic dysmotility of renal pelvis and ureter due to spinal cord injury.

## Case presentation

A 39-year-old British, Caucasian male, sustained T-4 complete paraplegia on 25 September 1988 when he fell off his motorbike. He was managing his bladder by penile sheath drainage. Intravenous urography, performed on 19 November 1988, was normal. In January 1991, he was prescribed trimethoprim 100 mg twice a day indefinitely. He was advised to perform intermittent catheterisation 2-3 times a day. In June 1991, this patient developed haematuria. On 28 February 1995, this patient developed fever, chills and vomiting. Blood urea was 23.7 mmol/L; creatinine: 334 umol/L; sodium: 135 mmol/L; potassium: 3.5 mmol/L. Ultrasound examination revealed marked hydronephrosis of right kidney and mild hydronephrosis of left kidney. Bilateral nephrostomy was performed on 07 March 1995.

Cystoscopy was carried out on 07 April 1995. Right ascending ureterogram showed normal ureter to pelvi-ureteric junction. A 7 Fr JJ stent was passed. Left ascending ureterogram showed stone just below pelvi-ureteric junction. Ureteroscopy was performed. Electrohydraulic lithotripsy was carried out and stone was partially fragmented. A 7 Ch JJ stent was passed into left kidney. On 16 June 1995, right JJ stent was removed. On 21 June 1995, extracorporeal shock wave lithotripsy of left ureteric calculus was carried out. On 09 August 1995, right antegrade pyelography was performed, which revealed dilated pelvicalyceal system with complete block at the pelvi-ureteric junction. Left ureteric stent was removed on 05 September 1995. Both nephrostomy tubes were removed on 25 November 1995.

Intravenous urography, performed on 20 March 1998, showed right hydronephrosis. This patient underwent right pyeloplasty on 08 May 1998. Right ureteric stent was removed on 30 June 1998.

**Figure 1:**
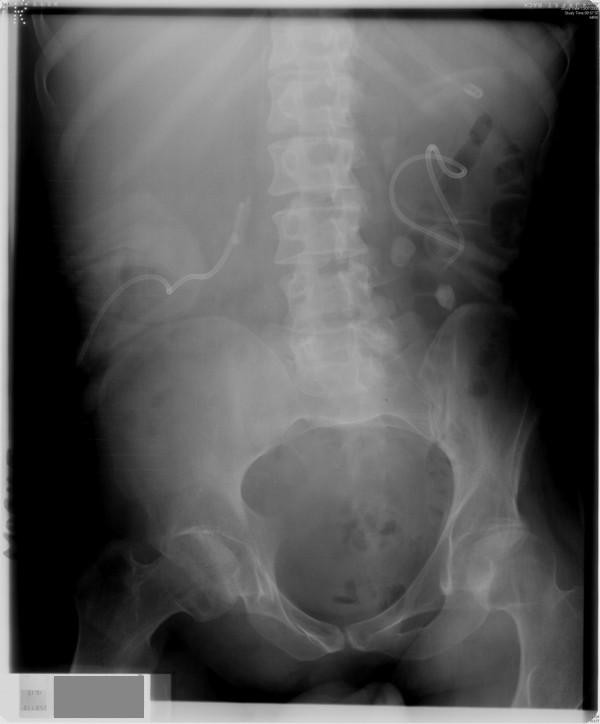
**X-ray of kidneys (13 July 2005) showed nephrostomy catheters in both kidneys**. Calculi were present in left renal pelvis and inferior calyx.

This patient became unwell on 29 August 2000. The scar of previous nephrostomy on left side had given way and there was discharge of pus. On 30 August 2000, intravenous urography showed prompt excretion of contrast by left kidney; normal left pelvicalyceal system, ureter and bladder. There was delay in the excretion of contrast by right kidney; forty minutes film showed dilated right calyceal system. Computed tomography of abdomen, performed on 30 August 2000, revealed 5 cm × 4 cm fluid collection just posterior to left kidney. Left renal outline appeared normal. Right hydronephrosis was noted. This patient was prescribed gentamicin and metronidazole. Follow-up computed tomography of upper abdomen was performed on 02 October 2000; this revealed marked resolution of left peri-renal abscess. A tiny 1 cm × 5 mm fluid collection remained just lateral to left psoas muscle. There was mild residual thickening of the peri-renal fascia. There was right-sided hydronephrosis. In July 2005, this patient developed urine infection and his General Practitioner prescribed cephalexin. On 10 July 2005, he was admitted to a local hospital with history of fever and rigors. He developed septicaemia and required mechanical ventilation. Ultrasound examination of abdomen, performed on 11 July 2005, revealed bilateral hydronephrosis and multiple stones in left kidney. On 12 July 2005, percutaneous nephrostomy was performed on both sides. This patient was transferred to spinal unit on 13 July 2005. X-ray of kidneys showed stones in left kidney (Figure 1). Tracheostomy was performed on 15 July 2005. His condition improved and he was weaned off ventilator. On 05 August 2005, extracorporeal shock wave lithotripsy of stones in left kidney was carried out. Shock wave lithotripsy was performed subsequently on 19 August 2005, 29 September 2005 and 23 November 2005.

On 10 January 2006, right nephrostomy did not drain urine. Nephrostogram revealed the tube to be lying outside pelvicalyceal system. The contrast entered perinephric tissue; therefore, the right nephrostomy tube was removed. Left nephrostogram showed that the contrast did not flow freely down left ureter. Intravenous urography, performed on 26 January 2006, showed right hydronephrosis due to pelvi-ureteric junction obstruction. There was dilatation of left pelvicalyceal system. On 31 January 2006, percutaneous right nephrostomy was performed. Since then right nephrostomy was anchored to skin with Percufix catheter cuff (Boston Scientific Corporation, One Boston Scientific Place, Natick, MA 01760-1537, USA).

MAG-3 renogram, performed on 06 February 2006, showed relative function of left kidney to be 71% and the right kidney 29%. There was normal uptake on the left and reduced uptake on the right at two minutes. Excretion was slow and sluggish from left kidney; however, excretion was diminished and poor from right kidney with the radioisotope activity gradually increasing with time. There was functionally significant obstruction within right kidney. The left kidney showed evidence of partial obstruction at the level of pelviureteric junction with preserved function.

**Figure 2:**
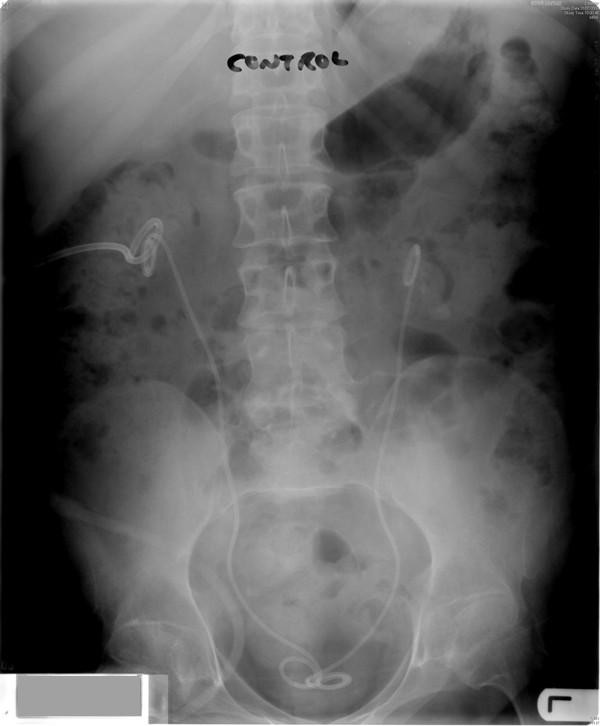
**X-ray of abdomen (31 July 2006) showed stents in both ureters**. Nephrostomy catheter was seen in right kidney. Left nephrostomy had been removed.

On 25 April 2006, cystoscopy showed small, contracted bladder. Left ureteric orifice was visualised. Ureteric catheter would go for one centimetre only. Even a Terumo guide wire could not be inserted. A Terumo guide wire was inserted through right ureteric orifice. Under fluoroscopy, right pelvi-ureteric junction was dilated with a balloon. On 26 April 2006, cystography was performed. Urinary bladder was of small capacity. There was no vesico-ureteric reflux. On 20 June 2006, left nephrostomy catheter was removed. Ureteric J stent was inserted through nephrostomy track and was placed in good position (Figure 2). On 06 March 2007, right nephrostomy tube was changed under fluoroscopy. On 11 May 2007, cystoscopy was performed. Both ureteric stents showed encrustation. Right ureteric stent was grasped and removed. Left ureteric stent was removed with difficulty. Concretions were present all over the stent. Retrograde pyelography showed dilated renal pelvis. A stent was inserted in left kidney. On 29 September 2007, cystoscopy was performed. Left ureteric stent was removed. It was not possible to insert a guidewire beyond L4/L3 level. Ureteroscopy was performed and a ureteric catheter was passed. Retrograde pyelography showed large dilated pelvis. Pus was drained through ureteric catheter. Ureteric stent was inserted. On 09 October 2007, exchange of right nephrostomy tube was performed. A 12 French pigtail catheter was inserted over guidewire and left on free drainage.

**Figure 3:**
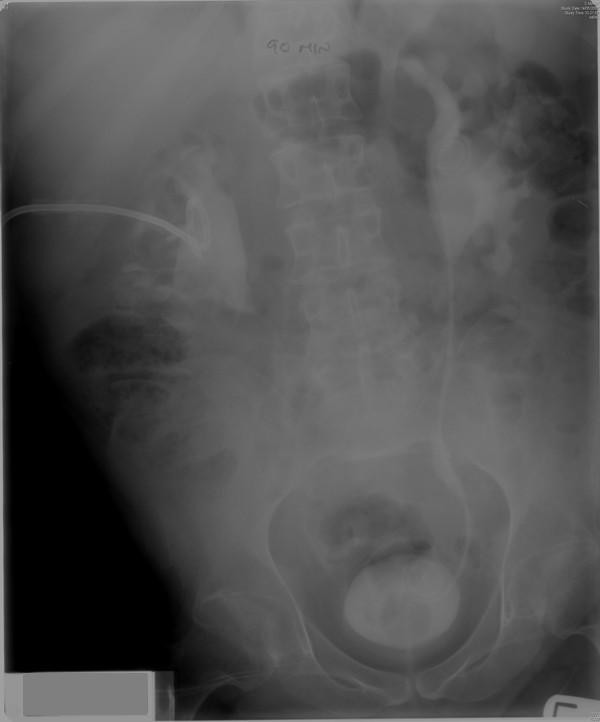
**Intravenous urography (14 May 2007) - 90 minutes film showed bilateral hydronephrosis**.

Intravenous urography, performed on 14 May 2007, showed bilateral hydronephrosis suggestive of bilateral pelviureteric junction obstruction (Figure 3). Left ureteric stent and right nephrostomy were present. CT of kidneys, performed on 12 November 2007, showed two opaque calculi in lower pole of left kidney. Left ureteric J stent was in situ. Right nephrostomy catheter was in situ. No opaque calculus was seen in the right kidney. There was a fragment of ureteric stent in the posterior cortex of the right kidney at the junction of middle and upper thirds.

**Figure 4:**
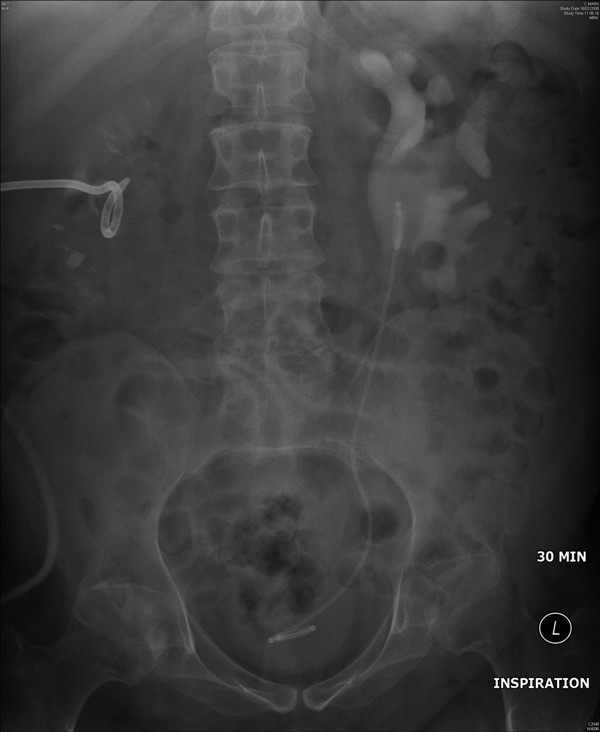
**Intravenous urography (18 February 2008) - 30 minutes film showed dilated left renal pelvis and clubbing of calyces**. Left ureteric stent and right nephrostomy catheter were present. Right nephrostomy catheter had not been clamped; therefore, urographic contrast drained straightaway from right kidney.

Intravenous urography, performed on 18 February 2008, showed a left sided JJ stent and a right nephrostomy tube in situ. A second tubular structure was seen close to the right nephrostomy tube. Appearances suggested a portion of tubing, which was of the same calibre as the left sided JJ stent. A number of calcific densities were seen in the region of the lower pole left kidney. No other urinary tract calcification was seen on the control film. The collecting systems of the left kidney were Duplex in nature and the left kidney was enlarged compared with the right. There was bilateral contrast excretion but again this was more marked on the left than the right. There was blunting of the minor calyces throughout the left kidney and prominence of the left renal pelvis suggesting previous pelviureteric junction obstruction (Figure 4). Very little anatomical detail was visible in the right kidney. The right ureter was not visualised but overall appearances did not suggest any obstruction. No useful contrast enhancement was seen within the bladder.

On 29 February 2008, cystoscopy was performed; left ureteric stent was removed. A 12-month stent was inserted in left ureter. Flexible ureteroscopy was performed on 28 March 2008. It was not possible to retrieve fragment of ureteric stent, which had been lying within right kidney. On 23 May 2008, right nephrostomy track was dilated to size 24 French. Flexible cystoscope was inserted. The fragment of ureteric stent was grasped and retrieved.

**Figure 5:**
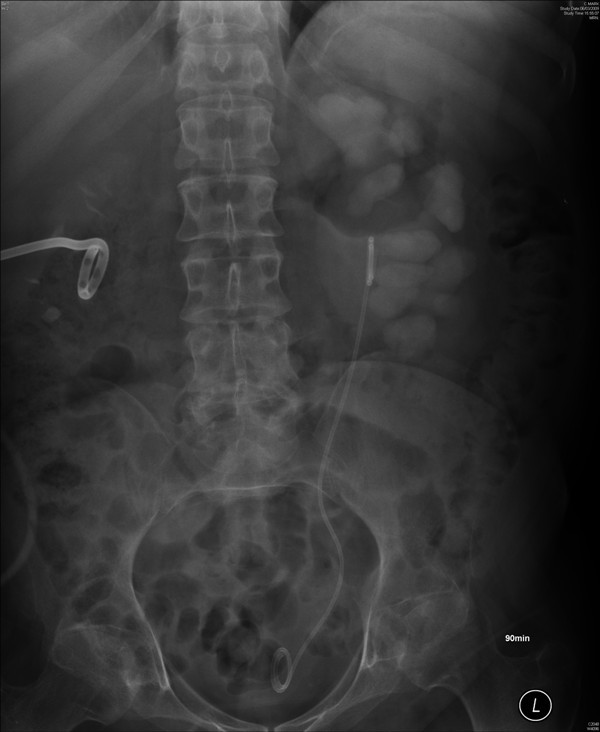
**Intravenous urography (06 March 2009) - 90 minutes film showed marked hydronephrosis on left side**. Left ureteric stent and right nephrostomy catheter were present. Right nephrostomy catheter had not been clamped; therefore, urographic contrast drained straightaway from right kidney.

Intravenous urography (IVU) was performed on 06 March 2009. IVU showed right nephrostomy tube and left double J stent in situ. The right nephrostomy tube had been exchanged but no other significant interval change was seen since the examination of 18 February 2008. In particular, there was no evidence of calcification seen in association with the left ureteric stent. There was bilateral excretion and the right kidney was shrunken and scarred compared with the left. The left kidney showed residual dilatation in the collecting systems. There was relatively poor drainage down into the bladder. The degree of dilatation in the left kidney had increased since the examination of 18 February 2008 (Figure 5). Appearances suggested some decrease in function of the left ureteric stent.

Microbiology of urine obtained from right nephrostomy on 06 March 2009 showed *Klebsiella oxytoca*, sensitive to gentamicin.

On 13 March 2009, left ureteric stent was removed and a Contour VL stent was inserted in left ureter.

Both kidneys were visualised in the summed images of MAG-3 renogram, which was performed on 19 March 2009. There was increasing tracer retention within both renal pelvis throughout the study. On the derived renogram curves (F-20), both kidneys showed moderate uptake of tracer. Drainage from both kidneys was sluggish with slight upward rising curves, suggesting underlying obstructions to both urinary systems. Some tracer however, was seen within the right nephrostomy, left ureter and bladder. The left kidney was contributing 49% and the right 51% of total renal function.

**Figure 6:**
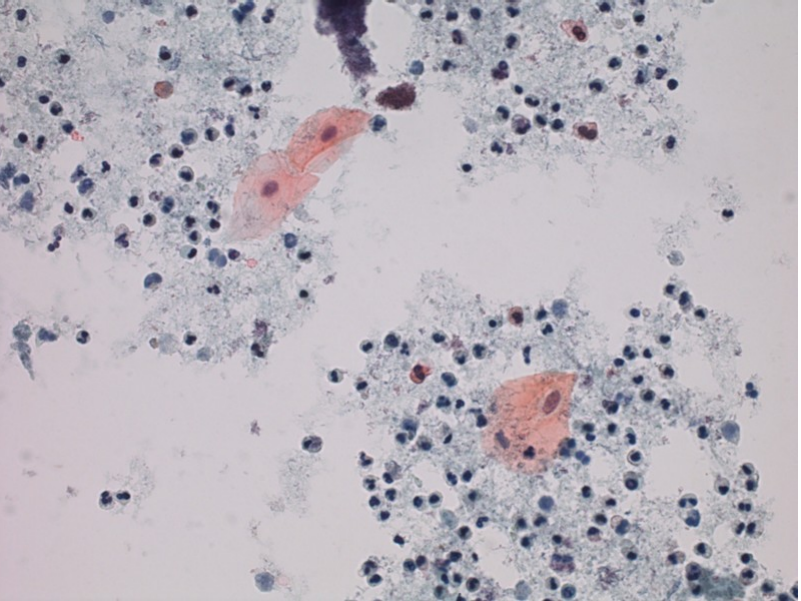
**Cytology of urine from nephrostomy, shows three benign squamous cells (large cells with abundant, pink-orange cytoplasm), with numerous inflammatory cells and inflammatory debris in the background**.

Cytology of urine from right kidney taken on 13 May 2009 showed large numbers of acute inflammatory cells, suggesting current acute urine tract infection. Benign epithelial cells were also present, many of which were squamous, suggesting the presence of squamous metaplasia (Figure 6). Occasional groups of cytologically bland urothelial cells were also present, but these could be explained by the presence of nephrostomy tube. No anucleate squames were present to suggest keratinising squamous metaplasia. There was no evidence of high-grade malignancy.

Microbiology of a swab taken from right nephrostomy site showed a heavy growth of coliforms on 28 April 2009. Currently, right kidney is drained by percutaneous nephrostomy and left kidney is drained by ureteric stent. This patient has indwelling urethral catheter drainage. He wears two leg bags and works full time.

## Discussion

Instead of external drainage of kidney by means of percutaneous nephrostomy, nephrovesical subcutaneous ureteric bypass has been performed in patients with ureteric obstruction due to inoperable malignancy [2,3]. Nephrovesical subcutaneous ureteric bypass consists of two subcutaneously connected 12 French polyurethane tubes, placed as a nephrostomy and cystostomy. This nephrovesical ureteric bypass is a simple, minimally invasive, and highly effective treatment for patients with hydronephrosis resulting from advanced oncologic disease. Patients gain a better quality of life due to increased independence and mobility during their final stages of life. Subcutaneous urinary diversion with a nephrovesical stent provides effective urinary drainage and may improve the quality of life of patients with malignant metastatic ureteral obstruction.

The Detour extra-anatomic stent (Mentor-Porges, UK) has also been used for permanent bypass of complete upper urinary tract obstruction [4]. This self-retaining expanded polytetrafluoroethylene-silicone tube is placed in the kidney using a percutaneous route, tunnelled under the skin, and sutured into the bladder to establish extra-anatomical urinary drainage. Preliminary data suggested that the Detour extra-anatomic stent offered a permanent and minimally invasive method to establish internalisation of urinary drainage to bypass complete ureteric obstructions for which conventional stenting had failed, open surgery had been tried and failed or was not considered feasible, and long-term nephrostomy drainage was not favoured.

When pyeloplasty is unsuccessful, a repeat open pyeloplasty is an option in neurologically intact individuals. Thomas and associates from Vanderbilt Children's Hospital, Nashville, Tennessee, USA [5], reviewed their experience with open dismembered pyeloplasty, with specific focus on the presentation and management of failed pyeloplasty in the pediatric population. Failure of pyeloplasty was most likely secondary to technical issues, including missed crossing vessels and dependency of the anastomosis. In this series, failed pyeloplasties did not respond well to balloon dilation, likely due to scar formation. These authors' current practice was to manage failures by open surgery, although endoscopic management by an incision might be an option. Braga and associates [6] compared retrograde endopyelotomy to redo pyeloplasty for the treatment of failed pyeloplasty in children. Retrograde endopyelotomy had a significantly lower success rate than redo pyeloplasty for correction of recurrent ureteropelvic junction obstruction after failed pyeloplasty in children.

Our patient with spinal cord injury and paraplegia developed bilateral hydronephrosis after he started managing his bladder by reflex voiding. In this patient, spinal cord injury resulted in neuropathic urinary bladder and neurogenic dysmotility of ureter and renal pelvis. Initially we performed right pyeloplasty and then balloon dilatation of right pelviureteric junction. Both procedures were unsuccessful in establishing satisfactory drainage of urine from right kidney. In hindsight, we recognised futility of these procedures, as neither of these procedures addressed the underlying pathology of neurogenic dysmotility of renal pelvis and ureter. In retrospect, we admitted our folly of performing these surgical procedures for treatment of hydronephrosis due to neurogenic dysmotility of pyeloureteral tract. Then, we adopted a pragmatic approach to the problem and relied upon percutaneous nephrostomy for drainage of right kidney and ureteric stent for drainage of left renal pelvis. At present, this patient has a size 14 Fr. pigtail catheter for nephrostomy. The nephrostomy is securely anchored to skin. The dressing is changed every Tuesday afternoon. The nephrostomy catheter is changed every six months. The patient has been coping with external drainage of kidney very well.

## Conclusion

We learn from this case the importance of preventing urological complications in patients with spinal cord injury. It is possible that regular intermittent catheterisations along with anticholinergic medication right from the time of rehabilitation might have prevented the series of urological complications, which occurred in this patient. Key components to successful management of external drainage of kidney in this patient are: [1] use of size 14 French pigtail catheter for long-term nephrostomy, [2] anchoring the catheter to skin to prevent accidental tug, [3] replacing the nephrostomy dressing once a week by the same team in order to provide continuity of care, and [4] changing nephrostomy catheter every six months by a senior Radiologist. This patient has been doing well and he is in full time employment as an expert web-designer.

## Patient's perspective

I have lived with nephrostomy drainage since July 2005, when I was taken into hospital with blockages in both kidneys. This was due to a large stone in my left kidney and restriction to my right ureter. This along with a chest infection, left me quite unwell so that I had to be sedated and ventilated for a few weeks. When I was taken off sedation, I discovered nephrostomy drainage to both kidneys and I had also been given a tracheostomy.

My life since the nephrostomy drainage was inserted has greatly improved and kidney function has increased. I feel much better now and I get far less UTI/kidney infections. In the past these have been regular occurrences and have caused lots of illness not to mention having time off work sick.

I still have one nephrostomy in the right ureter but the left has been removed for now although it may possibly be reinserted in the future if needed.

I do not mind having nephrostomy drainage as they have improved my wellbeing, which in turn has greatly improved my quality of life.

I attend Spinal Injuries Unit Outpatient Department one day a week to get the nephrostomy dressing changed and the tube cared for, this keeps the skin surrounding the insertion site in good condition and free from infection which could be a major problem if the skin breaks down, so attending on a regular basis is very important for my nephrostomy care.

On a personal note, the nephrostomy drainage does not really get in the way as to cause any major day to day problems, the only issue is time away from work to attend spinal injuries unit out patient department, but due to my condition being related to my disability (paraplegia), my employer has made reasonable adjustment to my job allowing me to have one afternoon a week off, this is a small problem to overcome when my quality of life has been improved so significantly.

## Competing interests

The authors declare that they have no competing interests.

## Authors' contributions

SV developed the concept and wrote the draft; GS performed pyeloplasty and balloon dilatation; PH performed percutaneous nephrostomiy, exchange of nephrostomy tubes, and antegrade stenting of left ureter; PH also reviewed medical images; BMS was the consultant in charge of patient; PM reported urine cytology; TO provided clinical care. All authors read and approved the final manuscript.

## Consent

Written informed consent was obtained from the patient for publication of this case report and accompanying images. A copy of the written consent is available for review from the journal's Editor-in-Chief.
